# Hybrid EEG–fNIRS-Based Eight-Command Decoding for BCI: Application to Quadcopter Control

**DOI:** 10.3389/fnbot.2017.00006

**Published:** 2017-02-17

**Authors:** Muhammad Jawad Khan, Keum-Shik Hong

**Affiliations:** ^1^School of Mechanical Engineering, Pusan National University, Busan, South Korea; ^2^Department of Cogno-Mechatronics Engineering, Pusan National University, Busan, South Korea

**Keywords:** brain–computer interface, hybrid EEG–fNIRS, mental task, classification, quadcopter control

## Abstract

In this paper, a hybrid electroencephalography–functional near-infrared spectroscopy (EEG–fNIRS) scheme to decode eight active brain commands from the frontal brain region for brain–computer interface is presented. A total of eight commands are decoded by fNIRS, as positioned on the prefrontal cortex, and by EEG, around the frontal, parietal, and visual cortices. Mental arithmetic, mental counting, mental rotation, and word formation tasks are decoded with fNIRS, in which the selected features for classification and command generation are the peak, minimum, and mean ΔHbO values within a 2-s moving window. In the case of EEG, two eyeblinks, three eyeblinks, and eye movement in the up/down and left/right directions are used for four-command generation. The features in this case are the number of peaks and the mean of the EEG signal during 1 s window. We tested the generated commands on a quadcopter in an open space. An average accuracy of 75.6% was achieved with fNIRS for four-command decoding and 86% with EEG for another four-command decoding. The testing results show the possibility of controlling a quadcopter online and in real-time using eight commands from the prefrontal and frontal cortices *via* the proposed hybrid EEG–fNIRS interface.

## Introduction

Brain–computer interface (BCI) or brain–machine interface (BMI) is a method of communication between brain and hardware by means of signals generated from the brain without the involvement of muscles and peripheral nervous system (Naseer and Hong, 2015b; Schroeder and Chestek, 2016). Although prosthetic devices utilize muscles or peripheral nerve signals (Ravindra and Castellini, 2014; Chadwell et al., 2016; Chen et al., 2016), brain signals are equally viable for provision of direct neural signals for interface purposes (Waldert et al., 2009; Quandt et al., 2012; Kao et al., 2014). A BCI, specifically, is an artificial intelligence system that can recognize a certain set of patterns generated by brain. The BCI promises as a platform to improve the quality of life of individuals with severe motor disabilities (Muller-Putz et al., 2015). The BCI procedure when acquiring control commands from the brain consists of five steps: signal acquisition, signal enhancement, feature extraction, classification, and control-interfacing (Nicolas-Alonso and Gomez-Gil, 2012).

The complicated surgical procedures performed for microelectrode implantation and establishment of BCI have been outstandingly successful in achieving control of robotic and prosthetic arms by means of neuronal-signal acquisition (Hochberg et al., 2006, 2012). These methods, however, are far from perfect options for BCI purposes, as they are all invasive and incur significant risks (Jerbi et al., 2011; Schultz and Kuiken, 2011; Rak et al., 2012; Ortiz-Rosario and Adeli, 2013).

The alternative non-invasive methods measure brain activities *via* either detection of electrophysiological signals (Li et al., 2010; Bai et al., 2015; Weyand et al., 2015) or determination of hemodynamic response (Bhutta et al., 2014; Ruiz et al., 2014; Hong et al., 2015; Naseer and Hong, 2015a; Weyand et al., 2015). Electrophysiological activity is generated by the neuronal firings prompted in the performance of brain tasks (Guntekin and Basar, 2016). The hemodynamic response is the increase of hemoglobin as a result of the neuronal firing that occurs when the brain performs an activity (Ferrari and Quaresima, 2012; Boas et al., 2014). The leading non-invasive BCI modalities in terms of cost and portability are electroencephalography (EEG) and functional near-infrared spectroscopy (fNIRS) (von Luhmann et al., 2015; Lin and Hsieh, 2016). The selection criterion for each modality is task dependent.

Electroencephalography has applications for active-, passive-, and reactive-type BCIs (Turnip et al., 2011; Zander and Kothe, 2011; Turnip and Hong, 2012; Urgen et al., 2013; Yoo et al., 2014). It is most widely employed with reactive-type tasks in the performance of which the brain output is generated in reaction to external stimulation. Commands are generated by detection of steady-state visually evoked potentials (SSVEP) and P300-based activations (Li et al., 2010, 2013; Turnip and Hong, 2012; Cao et al., 2014; Bai et al., 2015). fNIRS-based BCIs, meanwhile, are most commonly of the active type, which obtains brain activity output *via* user intentionality, independent of external events. For the purposes of fNIRS-based active BCIs, mostly mental (e.g., math, counting, etc.) and motor-related tasks (e.g., motor imagery) are selected (Naseer et al., 2014; Hong et al., 2015; Hong and Naseer, 2016). Although recent studies have shown the importance of fNIRS-based BCI for reactive and passive tasks (Hu et al., 2012; Santosa et al., 2014; Bhutta et al., 2015; Khan and Hong, 2015), active-type tasks are primarily used to increase the number of commands for this modality. The active-type BCI is preferred over the reactive BCI, as it allows a person to communicate with a machine at will. For both EEG and fNIRS, the drawback of increasing the number of active commands is the decrease in accuracy for BCI (Vuckovic and Sepulveda, 2012; Naseer and Hong, 2015a).

As a means of compensating for the accuracy reduction problem is the use of a single-brain signal acquisition modality, the hybrid BCI concept was proposed (Pfurtscheller et al., 2010). The design of a hybrid BCI entails the combination of either two modalities (at least one of which is a brain signal acquisition modality) or different brain signals (e.g., SSVEP and P300). The EEG–fNIRS-based hybrid BCI has been reported to enhance classification accuracy (Fazli et al., 2012; Putze et al., 2014; Tomita et al., 2014) and increase the number of commands (Khan et al., 2014). Classification accuracy can be improved by simultaneously decoding EEG and fNIRS signals for the same activity and combining the features. The number of active commands can be increased by decoding brain activities from different brain regions (e.g., motor tasks for EEG and mental tasks for fNIRS). However, for these cases, the reported window size using fNIRS for optimal classification is around 10 s (Tomita et al., 2014). The problem of window size reduction and others relevant to real-time/online BCI applications require further research. Table 1 summarizes the most recent work (Kim et al., 2014; Bai et al., 2015; Combaz and Van Hulle, 2015; Hortal et al., 2015; Ma et al., 2015; Naseer and Hong, 2015a; Ramli et al., 2015; Yin et al., 2015) in terms of command number, accuracy, and window size as those parameters relate to robotic-control applications.

**Table 1:** **Comparison of our proposed method with recent electroencephalography (EEG)-based work on command generation, accuracy, and window size**.

Reference	Brain area	Activity	Brain–computer interface (BCI) type	Modality	Application	Commands	Accuracy (%)	Window size
Kim et al. (2014)	Complete brain	Eye movement	Active	EEG + Eye tracker	Quadcopter control	8	91.67	5 s
Bai et al. (2015)	Complete brain	Motor imagery and P300	Active + reactive	EEG	Opening, closing, selection of files in Internet Explorer	9 (can achieve 50)		4 s window for motor imagery and 600 μs for P300
Hortal et al. (2015)	Motor and parietal	Mental imagination	Active	EEG + EOG	Robotic arm control for pick and place task	6	Task 1:71.13 and Task 2:61.51	0.5 s to synchronize output to brain–machine interface
Naseer and Hong (2015a)	Prefrontal and motor cortex	Mental arithmetic, mental counting and motor imagery	Active	Functional near-infrared spectroscopy	Decoding answers to four-choice questions	4	73.3	2–7 s
Ma et al. (2015)	Parietal and occipital	P300 and eyeblink	Reactive + active	EEG + EOG	Mobile robot control	9	87.3 for average of 5 trials	~1.6 s
Combaz and Van Hulle (2015)	Whole brain	P300 and steady-state visually evoked potentials (SSVEP)	Reactive	EEG	Applications to locked-in patients option selection	12	Maximum achieved >95	200 μs before stimulation to 800 μs after stimulation for experiment 1
Ramli et al. (2015)	Motor and occipital	Eye gaze	Reactive	EEG + EOG	Application to BCI applications (wheelchair control)	6	97.88	0.5 s
Yin et al. (2015)	Parietal and occipital cortex	P300 and SSVEP	Reactive	EEG	Speller paradigm with applications to BCI systems control	Up to 64 commands	95.18	
The proposed method	Frontal	Mental task + eye movement	Active	NIRS + EEG	Applications to quadcopter control	8	76.5% for NIRS and 86% for EEG	1 s for EEG and 2 s for NIRS

In the present BCI research, we decoded eight active commands using signals from the frontal and prefrontal cortices. Four tasks (mental math, mental counting, word formation, and mental rotation) were decoded using fNIRS, and four eye movement signals (up/down eye movement, left/right eye movement, twice or three times eyeblinks) were decoded using EEG. In the fNIRS classification and generation of commands, a 0- to 2-s window was used, whereas in the case of EEG, a 1-s window was used. The commands thus generated were used to update a quadcopter’s movement coordinates (six movements and start/stop). Revealing the obtained results briefly, the signal mean, peak, and minimum-value features obtained using oxyhemoglobin data in 0–2 s window provided 76.5% accurate classification. For EEG, signal peak and number of peaks achieved 86% accurate results. The testing of the drone in an arena showed the possibility of quadcopter control using eight-brain commands from the frontal cortex. To the authors’ best knowledge, this is the first fNIRS study to decode and classify brain activity in 0–2 s window. Also, this is the first study to decode four commands from the prefrontal cortex using fNIRS. Moreover, this work shows the first hybrid EEG–fNIRS-based decoding of eight active commands from the frontal and prefrontal cortices.

## Materials and Methods

### Subjects

A total of 10 healthy adults were recruited (all right-handed males; mean age: 28.5 ± 4.8). Right-handers had been sought in order to minimize any variations in the electrophysiological and hemodynamic responses due to the hemispheric dominance difference. None of the selected participants had participated in any previous brain signal acquisition experiment, and none had a history of any psychiatric, neurological, or visual disorder. All of them had normal or corrected to normal vision, and all provided a written consent after having been informed in detail about the experimental procedure. Experiments with fNIRS and EEG were approved by the Institutional Review Board of Pusan National University, and they were conducted in accordance with the ethical standards encoded in the latest Declaration of Helsinki.

### Electrode/Optode Placement

**Figure 1:**
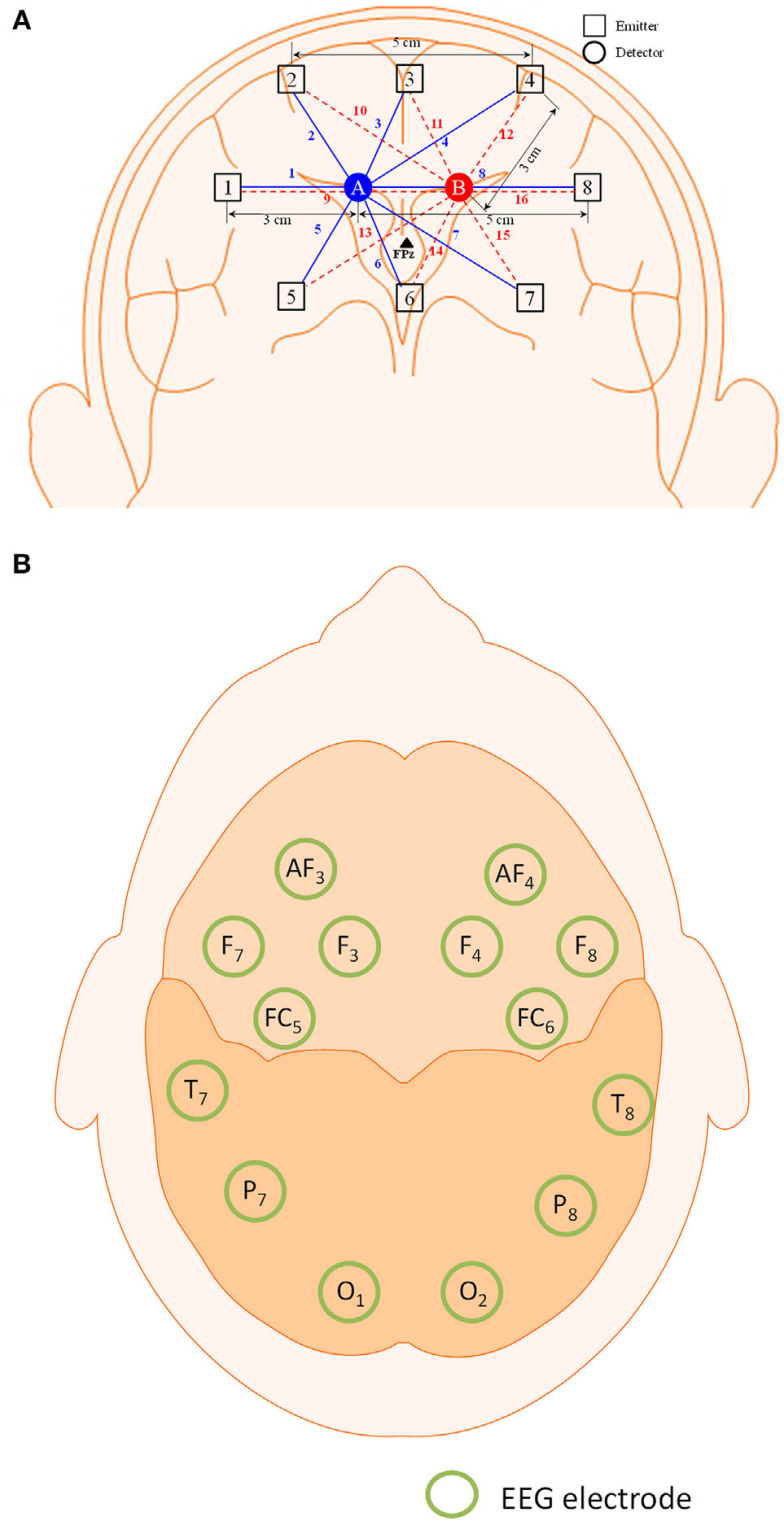
**Configuration of optodes and electrodes for hybrid functional near-infrared spectroscopy–electroencephalography (fNIRS–EEG) experiment**. **(A)** 16-channel fNIRS with 2 detectors and 8 emitters in the prefrontal brain region and **(B)** 14-electrode configuration of the Emotiv EEG headset.

The frequency domain system ISS Imagent (ISS Inc., USA) was used for the signal acquisition. A total of eight sources and two detectors, making a combination of 16 channels, were positioned around the prefrontal cortex. The FPz location was positioned between the two detectors. The Emotiv EEG headset (Emotiv Epoc, USA) was used to acquire the EEG signals. The electrodes/optodes were positioned on the head according to the International 10–20 system (Jurcak et al., 2007). The electrode and optode placement is illustrated in Figure 1.

### Experimental Procedure

The experimental procedure consisted of two sessions: training and testing. The subjects were trained to perform eight tasks detected simultaneously by EEG and fNIRS, after which the recorded brain activities were tested using real-time/online analysis.

#### Training Session

For the training session, the subjects were seated in a comfortable chair and told to relax. A computer monitor was set up approximately 70 cm in front of the subjects. The session began with a resting period of 2 min to establish a data baseline. After the resting period, the screen cued the participants to perform one of eight specific tasks. The tasks were as follows:

- *Mental counting*: counting backward from a displayed number;
- *Mental arithmetic*: subtraction of two-digit numbers from three-digit numbers in pseudo random order (e.g., 233 − 52 = ?, ? − 23 = ?);
- *Mental rotation*: visualization of the clockwise rotation of a displayed stationary object (i.e., a cube);
- *Word formation*: formation of five scrambled words (e.g., “lloonab”), the first letter of which is shown as a cue (e.g., “B”);
- *Two eyeblinks*: blinking twice within 1 s window;
- *Three eyeblinks*: blinking thrice within 1 s window;
- *Up/down eye movement*: movement of both eyes in the up or down direction within 1 s window;
- *Left/right eye movement*: movement of both eyes in the left or right direction.

**Figure 2:**
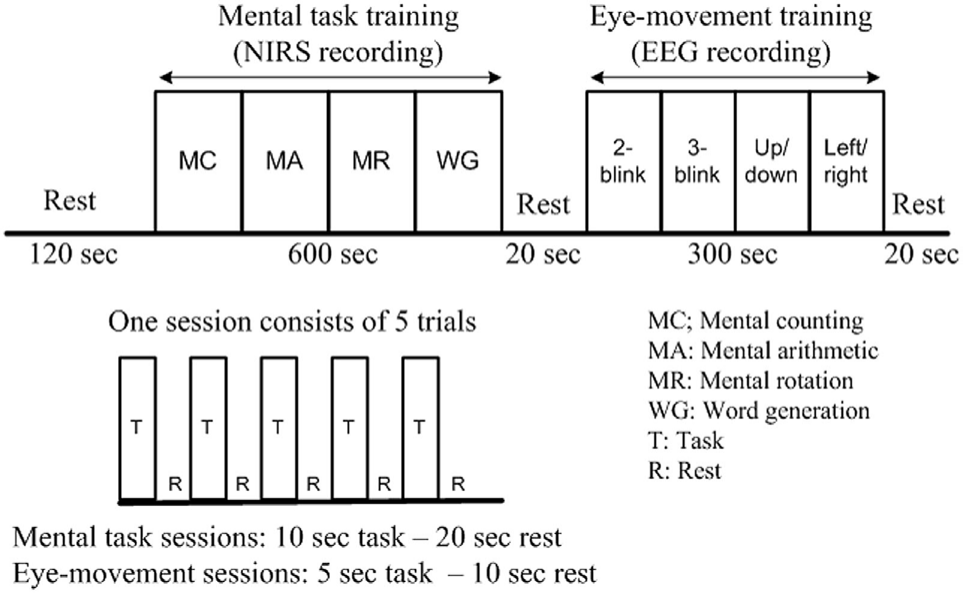
**Experimental paradigm of a training session (per subject)**. After the initial 2-min rest, each functional near-infrared spectroscopy recording block consists of five 10-s activations and five 20-s rests, while each electroencephalography block consists of five 15-s tasks and five 10-s rests. The total duration of the experiment is 17 min.

The mental tasks were recorded mainly using fNIRS, as the previous work (Weyand et al., 2015) has shown its utility for high-accuracy detection of the above-noted tasks. The eye movement tasks were recorded principally using EEG, as the Emotiv EEG head set, as noted earlier, is commercially available as a system for detection of various facial movements and motor signals. The training session was divided into two parts: mental task training and eye movement training. In the first part, the subjects were trained for mental arithmetic, counting, rotation, as well as word formation tasks. Each task consisted of five 10-s trials separated by a 20-s resting session. In the second part of the training session, the subjects were instructed to move their eyes according to the cue given. Each trial in this case was 5 s in duration, and the resting period was 10 s. Details on the experimental paradigm and data recording sequence are provided in Figure 2.

#### Testing Session

As part of the testing session, the training data were used to test the movement of a quadcopter (Parrot AR drone 2.0, Parrot SA., France). Specifically, the eight commands recorded during the training session were used to navigate the quadcopter in an open arena. The data were translated into commands and the subjects were asked to move the quadcopter in a rectangular path.

### Signal Acquisition and Processing

The data for both modalities (EEG and fNIRS) were independently processed and filtered to acquire the desired output signals. In both cases, band-pass filtering was used to remove physiological noise from the acquired signals.

#### fNIRS Signal Processing

The frequency domain fNIRS system used two wavelengths (690 and 830 nm) to determine the changes in the concentration of hemoglobin. The sampling rate of 15.625 Hz was used to acquire the data. The modified Beer-Lambert law (Baker et al., 2014; Bhatt et al., 2016) was utilized to convert the data into concentrated changes of oxy- and deoxy-hemoglobin (ΔHbO and ΔHbR):

(1) $$A(t;\lambda )=\ln\frac{I_{\text{in}}(\lambda )}{I_{\text{out}}(t;\lambda )}=\alpha (\lambda )\times c(\lambda )\times l\times d(\lambda )+\eta ,$$

(2) $$\left[\begin{array}{c} \Delta c_{\text{HbO}}(t)\\ \Delta c_{\text{HbR}}(t) \end{array}\right]={\left[\begin{array}{cc} \alpha_{\text{HbO}}(\lambda_{1}) & \alpha_{\text{HbR}}(\lambda_{1})\\ \alpha_{\text{HbO}}(\lambda_{2}) & \alpha_{\text{HbR}}(\lambda_{2}) \end{array}\right]}^{-1}\left[\begin{array}{c} \Delta A(t;\lambda_{1})\\ \Delta A(t;\lambda_{2}) \end{array}\right]\frac{1}{l\times d(\lambda )},$$

where *A* is the absorbance of light (optical density), *I*_in_ is the incident intensity of light, *I*_out_ is the detected intensity of light, α is the specific extinction coefficient in µM^−1^ cm^−1^, *c* is the absorber concentration in micromolars, *l* is the distance between the source and the detector in centimeters, *d* is the differential path-length factor, and η is the loss of light due to scattering.

The data were first preprocessed to remove physiological noises related to respiration, cardiac, and low-frequency drift signals. In order to minimize the physiological noise due to heart pulsation (1–1.5 Hz for adults), respiration (approximately 0.4 Hz for adults), and eye movement (0.3–1 Hz), the signals were low-pass filtered using a fourth-order Butterworth filter at a cutoff frequency of 0.15 Hz. The low-frequency drift signals were minimized from the data using a high-pass filter with a cutoff frequency of 0.033 Hz (Kamran and Hong, 2014; Bhutta et al., 2015; Hong and Santosa, 2016).

#### EEG Signal Processing

The 14-channel EEG data were acquired at a sampling rate of 128 Hz. The α*-*, β*-, Δ-*, and θ-bands, acquired by band-pass filtering between 8 and 12 Hz, 12 and 28 Hz, 0.5 and 4 Hz, and 4 and 8 Hz, respectively, enabled isolation of the electrodes corresponding to the eye movement activities (Lotte et al., 2007; Ortiz-Rosario and Adeli, 2013; Ma et al., 2015).

### Channel Selection

Several channels were activated for both EEG and fNIRS. Proper channel selection is essential to the high-accuracy generation of commands. Previous work has employed *t*-value-based approaches (Hong and Nguyen, 2014; Hong and Santosa, 2016), bundled-optode-based approaches (Nguyen and Hong, 2016; Nguyen et al., 2016), and channel-averaging approaches (Khan and Hong, 2015; Naseer and Hong, 2015a). Other studies alternatively have employed their own algorithms, for instance, independent component analysis, etc. (Hu et al., 2010; Kamran and Hong, 2013; Santosa et al., 2013). We used the following criteria for selection of fNIRS and EEG channels.

#### fNIRS Channel Selection

For fNIRS channel selection, we calculated the peak (max) value of ΔHbO in the baseline and in the first trials of the mental arithmetic, mental counting, mental rotation, and word formation tasks, respectively. If the difference between the max value of the trial and the baseline value was positive, the channel was selected for classification; if neutral or negative (equal to or less than zero), it was discarded.

#### EEG Channel Selection

In case of EEG, we measured the power spectrum for each channel. The selected channels were those in which the signal power corresponding to the eyeblink and movement tasks was significant. Mostly the channels near the frontal brain region were active in this case.

### Feature Extraction and Classification

In order to generate commands, we first extracted the relevant features for classification. We selected signal peak and signal mean as features as, according to the literature (Khan and Hong, 2015), they provide better performance for fNIRS-based BCI systems. Also, in consideration of a recently reported possibility of an initial fNIRS signal dip (Hong and Naseer, 2016; Zafar and Hong, 2017), we added a minimum (min) signal value as a feature. We also investigated the possibility of minimizing the time for command generation by means of 0–1, 0–1.5, and 0–2 s windows.

For EEG signals, following channel selection we selected the signal mean and number of peaks as features for command generation. In this case, we used a moving window of 1 s to extract the relevant feature values.

For both modalities, MATLAB^®^-based functions were used to calculate the features of the mean, peak, min, and number of peaks. For offline processing, the extracted features were rescaled between 0 and 1 by the equation

(3) $$a^{\prime }=\frac{a-\text{min}\;a}{\text{max}\;a-\text{min}\;a},$$

where *a* ∈ *R^n^* represents the feature value, *a*′ is the rescaled value between 0 and 1, max *a* denotes the largest value, and min *a* indicates the smallest value. These normalized feature values were used in a four-class classifier for training and testing of the data. We used linear discriminant analysis (LDA) to classify the signals for EEG and fNIRS, as, in one of our previous studies, we found it to be faster than support vector machine (Khan and Hong, 2015).

For our case, *x_i_* ∈ *R*^2^, where, for fNIRS, *i* denotes the classification class, μ*_i_* is the sample mean of class *i*, and μ is the total mean over all of the samples *l*. That is,

(4) $$\mu_{i}=\frac{1}{n_{i}}\sum_{x\in \text{class}\;i}\;x,\mu =\frac{1}{n}\sum_{l}x_{l},$$

where *n_i_* is the number of samples of class *i* and *n* is the total number of samples. The optimal projection matrix *V* for LDA that maximizes the following Fisher’s criterion is

(5) $$J\text{(}V\text{)}=\frac{\det\text{(}V^{T}S_{\text{B}}V\text{)}}{\det(V^{T}S_{\text{W}}V)},$$

where *S*_B_ and *S*_W_ are the between-class scatter matrix and the within-class scatter matrix, respectively, given by

(6) $$S_{\text{B}}=\sum_{i=\;1}^{m}n_{i}(\mu_{i}-\mu ){\left(\mu_{i}-\mu \right)}^{T},$$

(7) $$S_{\text{W}}=\sum_{i=\;1}^{m}\sum_{x_{l}\in \text{ class}\;i}(x_{l}-\mu_{i}){\left(x_{l}-\mu_{i}\right)}^{T},$$

where the total number of classes is given by *m*. Equation 5 was treated as an eigenvalue problem in order to obtain the optimal vector *V* corresponding to the largest eigenvalue. In the case of offline testing, 10-fold cross-validation was used to estimate the classification accuracy (Lotte et al., 2007; Hwang et al., 2013; Ortiz-Rosario and Adeli, 2013).

### Control Scheme for Quadcopter

**Figure 3:**
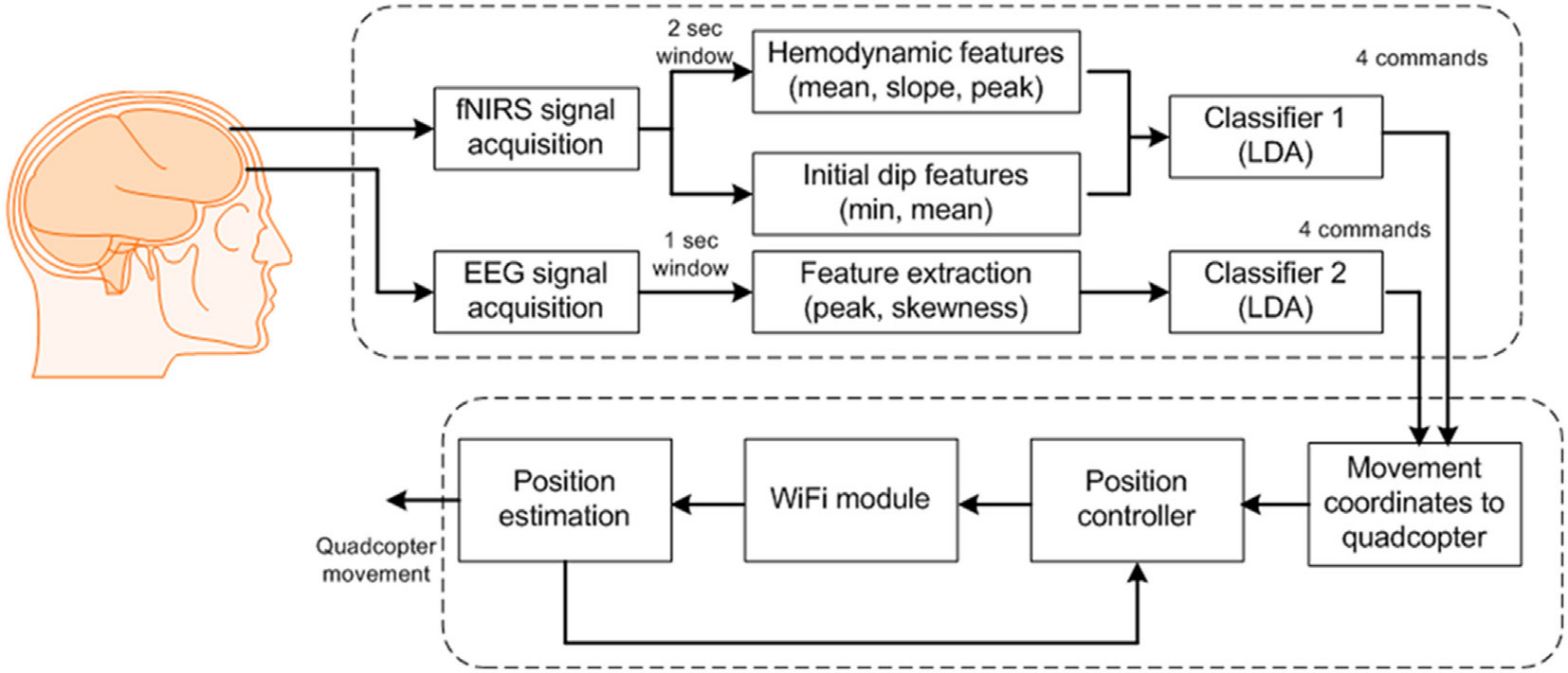
**Block diagram of the proposed brain–computer interface scheme for generation of eight commands**.

For control of the quadcopter, we formulated eight commands for classification: up/down movements, clockwise/counterclockwise rotations, forward/backward movements, and start/stop. After classification, we updated the quadcopter’s movement coordinates by Wi-Fi communication. The quadcopter has navigated using the transmitted commands. Figure 3 provides a block diagram of the BCI scheme for quadcopter control.

## Results

**Figure 4:**
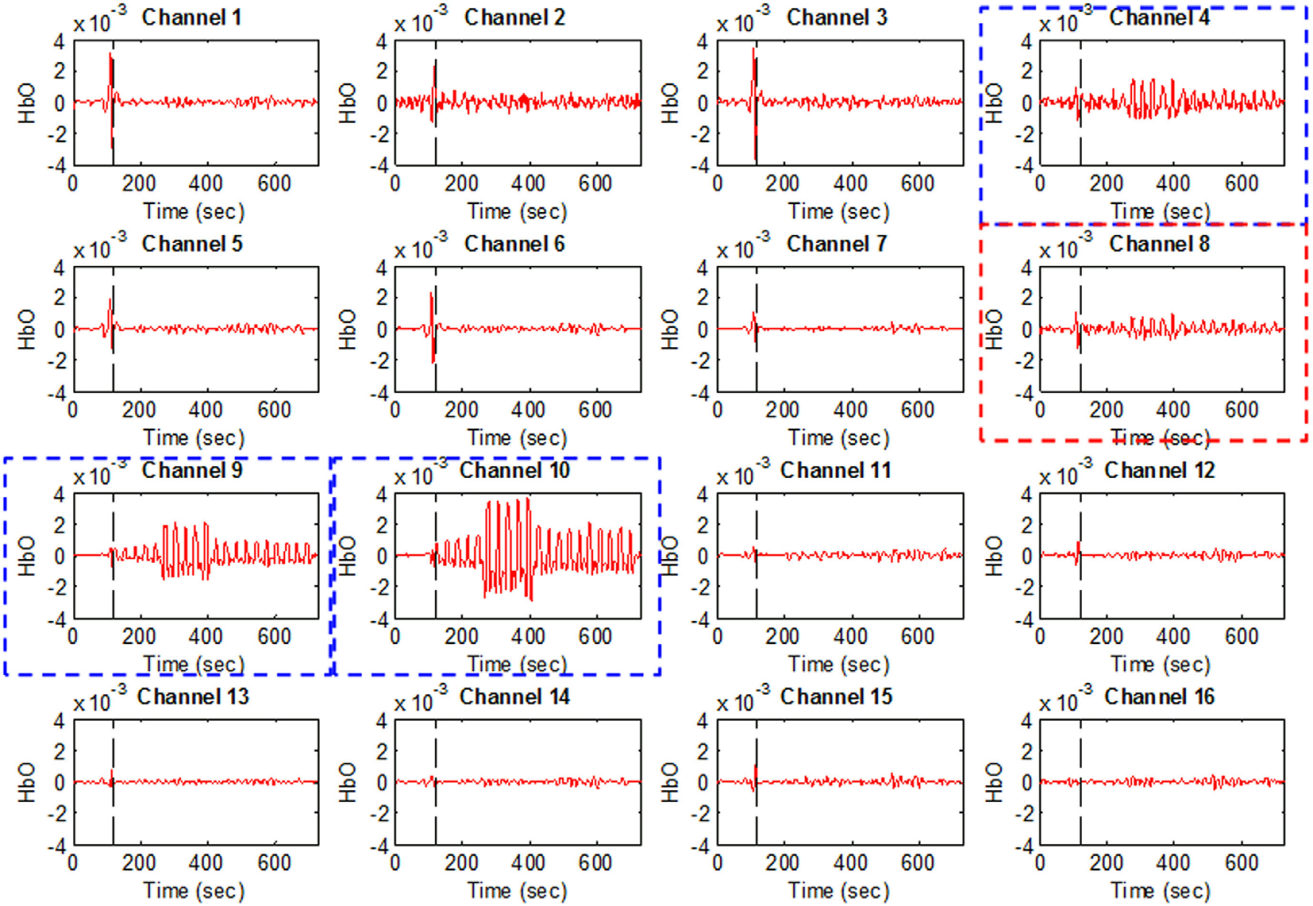
**HbO examples for Figure 1A (Subject 2)**. Channels 4, 9, and 10 were selected as active channels by the proposed method, but channel 8 was not (even if it was identified as such by the *t*-value analysis).

Figure 4 plots Subject 2’s ΔHbO values for all 16 channels and four activities. It can be seen that not all of the channels were active when performing a brain activity. However, for all four of the mental tasks, the activation pattern appears in the same channels.

The plots in Figure 4 serve to emphasize the necessity of selecting proper channels for distinguishing of brain activities. As per our channel-selection criterion, we subtracted the max value in the baseline from the max value of the first trial. Accordingly, channels 4, 9, and 10 were selected for Subject 2, whereas channel 8 was not, due to having a higher value of baseline. We intended to identify different brain channels for different activities; therefore, as per our criterion, the subtraction of the first trial for each activity can identify different channels. However, in this case, for all subjects selected, the common channels were activated as corresponding to the mental tasks.

**Figure 5:**
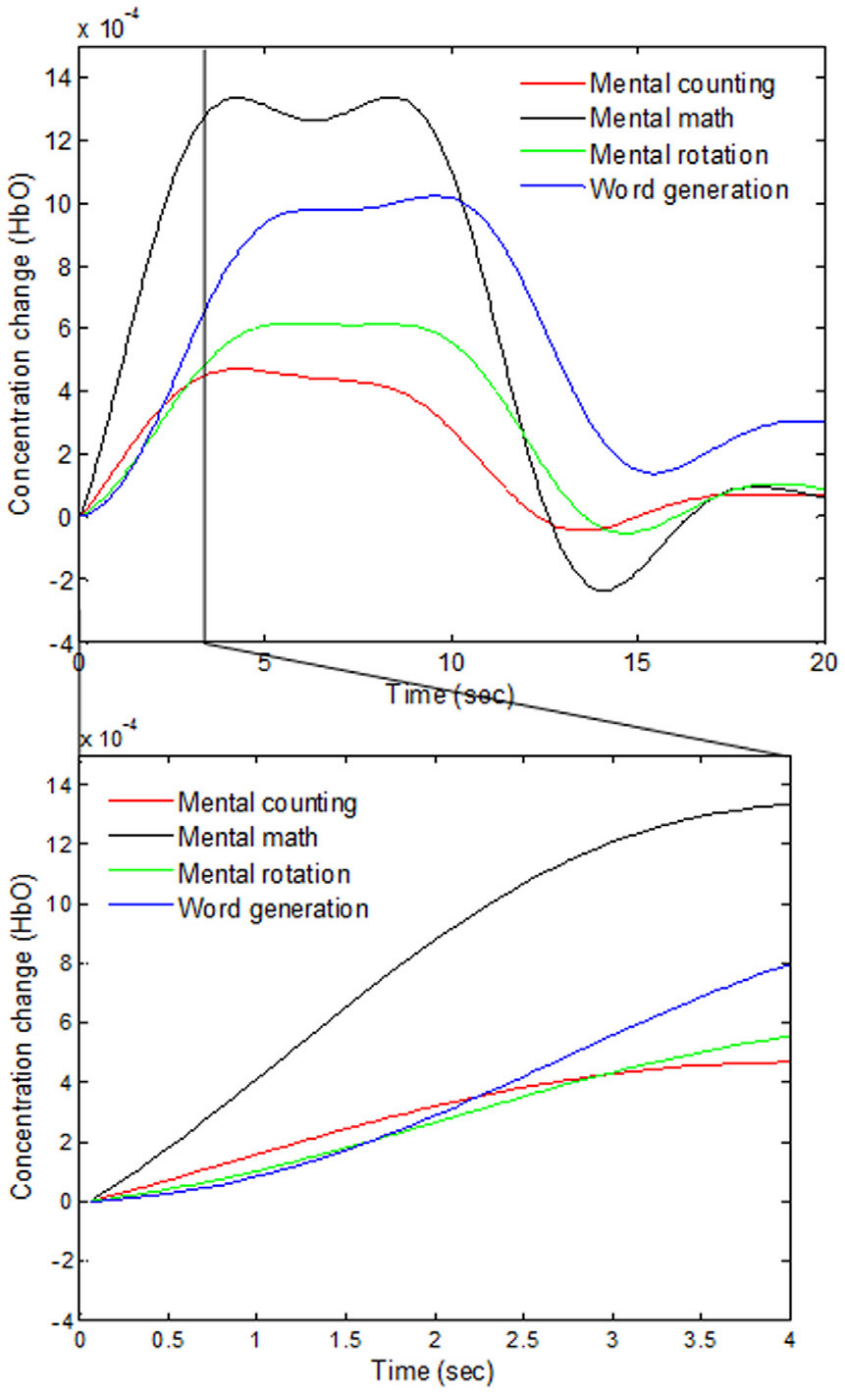
**Comparison of the averaged HbOs of four mental tasks and the magnified responses during 0–4 s window**.

As various windows sizes have been used for detection of fNIRS features in different studies (Utsugi et al., 2008; Luu and Chau, 2009; Power et al., 2010; Naseer and Hong, 2013; Schudlo et al., 2013; Schudlo and Chau, 2015; Weyand and Chau, 2015; Weyand et al., 2015; Naseer et al., 2016a,b), we intended to minimize the window size applicable to BCI applications. We therefore selected 0–0.5, 0–1, 0–1.5, and 0–2 s windows for feature acquisition and investigated both hemodynamic and initial dip features to acquire the best window size for reduced computation time. The signals of Subject 2 as averaged over all of the trials in the reduced window are plotted in Figure 5.

**Figure 6:**
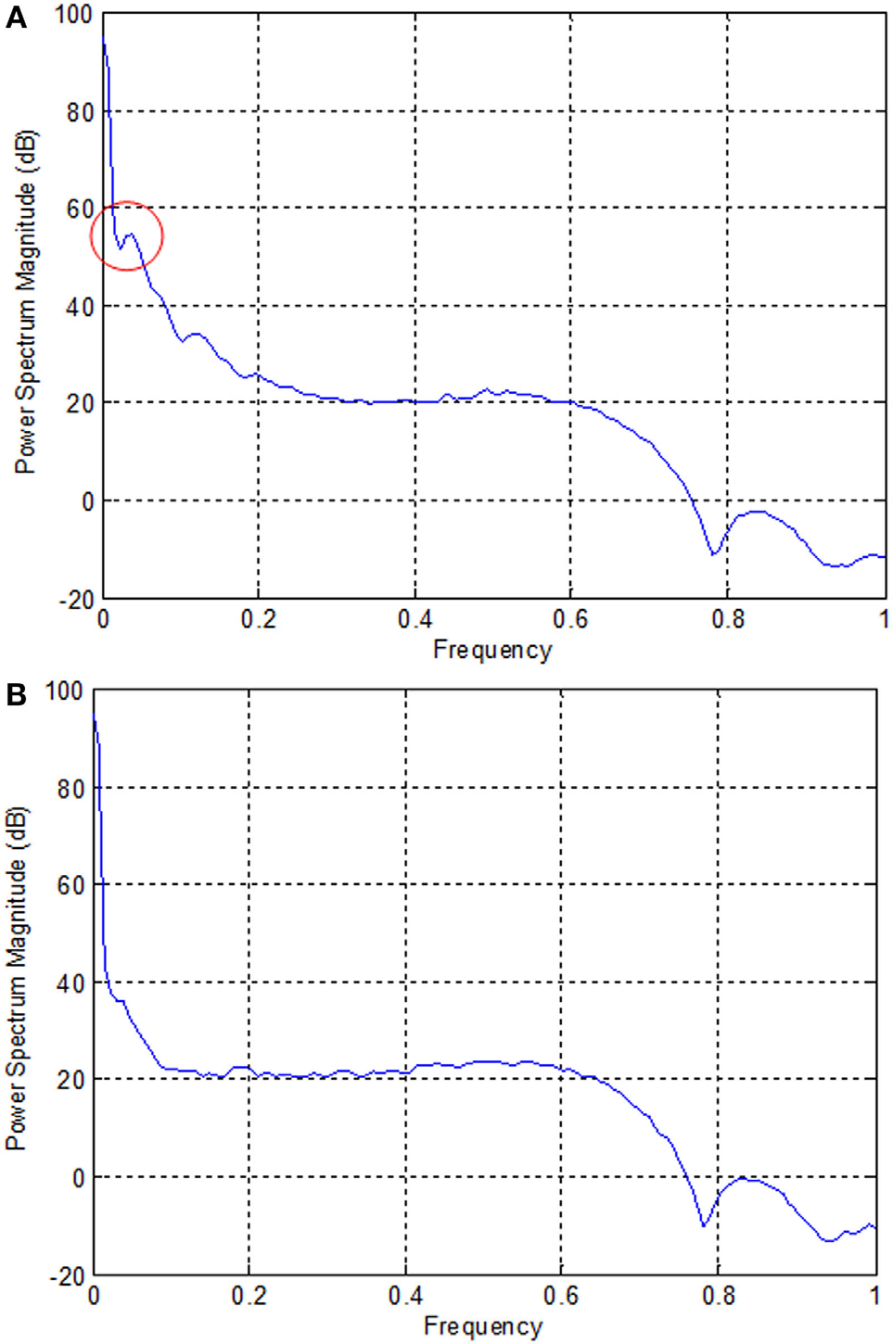
**Normalized power spectra of electroencephalography (Subject 2)**. **(A)** F3 electrode and **(B)** P7 electrode. The local peak in the red circle in **(A)** corresponds to the eye movement task measured by the F3 electrode.

In the case of EEG, we examined the power spectrum in order to identify the activated channels. The F3, F4, O1, and O2 regions were most active. We selected the channel showing the highest power corresponding to the eye movement task. Figure 6 plots the normalized power spectrum of the selected channel for Subject 2.

The accuracies obtained for fNIRS are shown in Table 2. The accuracies achieved using EEG for the selected channels are shown in Table 3.

**Table 2:** **Classification accuracies of four functional near-infrared spectroscopy window sizes (based upon the mean, peak, and minimum values of ΔHbO)**.

Subjects	Window size 0–0.5 s	Window size 0–1 s	Window size 0–1.5 s	Window size 0–2 s
1	65	70	70	75
2	80	85	90	95
3	80	80	85	85
4	50	55	60	65
5	85	85	90	95
6	70	70	70	75
7	55	60	65	70
8	65	60	65	70
9	60	65	65	70
10	50	55	60	65

Mean	66 ± 12.6	68.5 ± 11.5	72 ± 11.8	76.5 ± 11.3

**Table 3:** **Electroencephalography accuracies of selected electrodes**.

Subjects	Electrodes selected	Accuracy (%)
1	F3	100
2	F3	95
3	F3	100
4	AF3	90
5	F7	75
6	F3	75
7	F4	75
8	F3	80
9	F7	80
10	F3	90

Mean		86 ± 10.2

**Figure 7:**
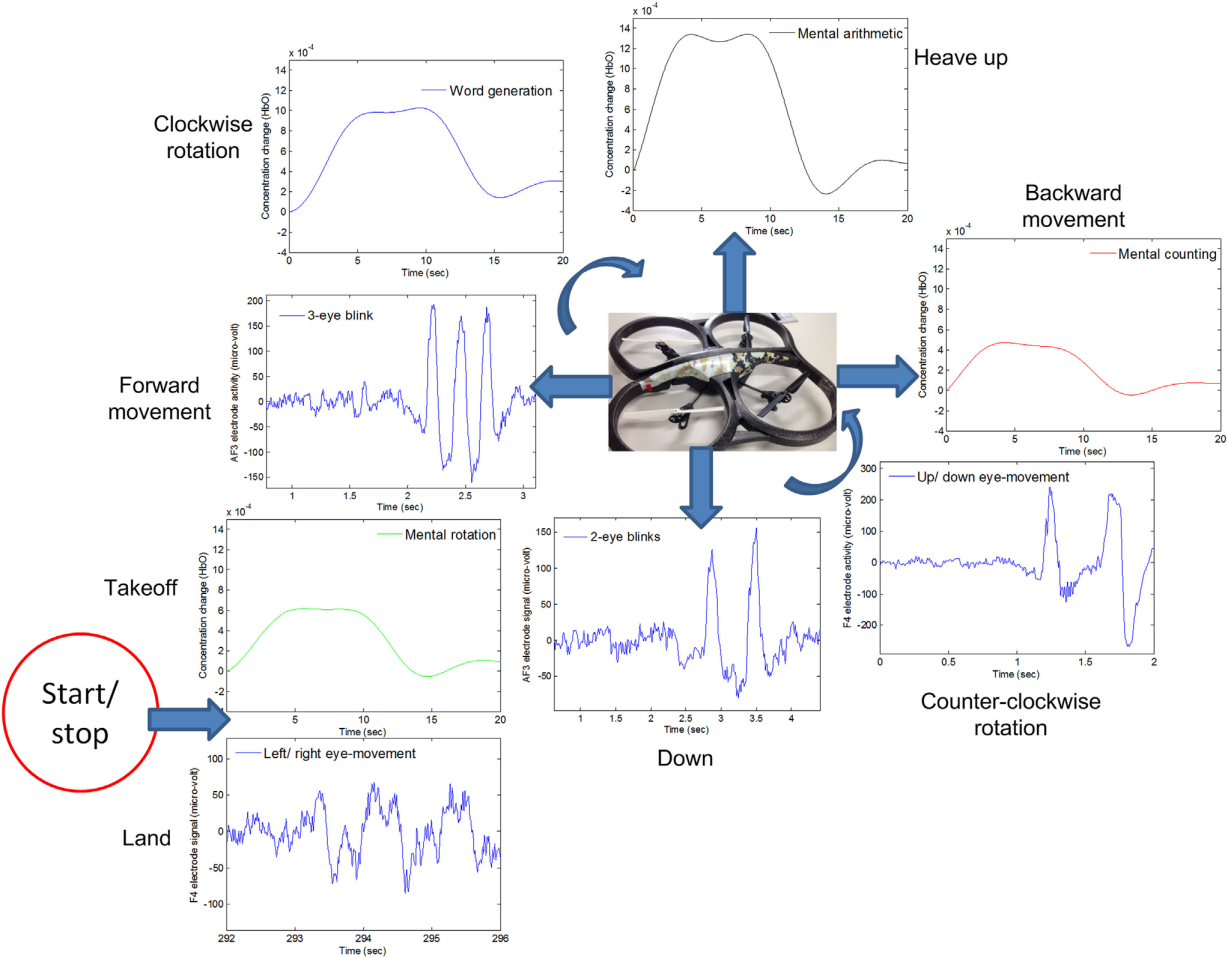
**The quadcopter control scheme based on electroencephalography and functional near-infrared spectroscopy signals**.

For real-time/online testing, we associated each activity with quadcopter movement. The associated activity for each is shown in Figure 7.

We associated opposite movements with EEG/fNIRS activities; for example, if forward movement was associated with EEG signals, backward movement was associated with fNIRS signals. This was done to ensure safety from the quadcopter and anyone in the area. This scheme has benefits in any case, as, if a command is misclassified/misinterpreted, a command with the second modality can be generated to countermove the misclassified movement. As per Figure 7, when EEG was used for one motion, fNIRS was used for its counter motion. This selection reflected EEG’s demonstratively higher accuracy for most of the subjects.

**Figure 8:**
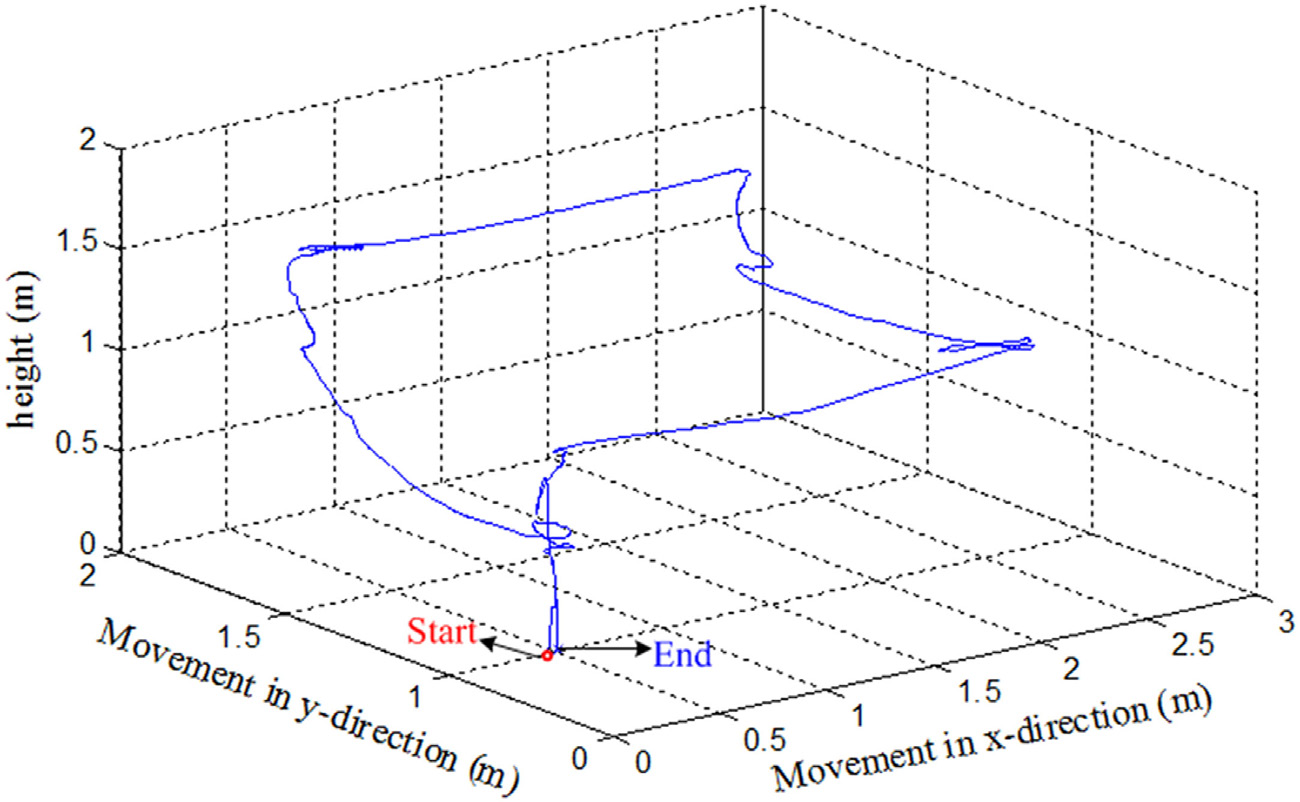
**An example of trajectories of the quadcopter in the 3D space (Subject 2)**.

We have tested the movement of the quadcopter in an arena. The subjects were asked to move the quadcopter in a rectangular path. They were asked to land the quadcopter near to the takeoff position. After take off, the subjects were informed to move the drone almost 3 m in forward direction, then 2 m to the left. The subjects had to increase the height by almost 0.5 m when reaching the left corner. After increasing the height, the subjects were to move the quadcopter backward 3 m and then 2 m to the right to reach the takeoff spot. After reaching the final position, the subjects were asked to land the drone. The path followed by the drone from Subject 2 is shown in Figure 8.

Since the drone requires quick response commands to maneuver, it can be clearly seen that the path was not properly followed. The subject had to adjust the path to the desired path to reach the final position. This is due to the delay in command generation and transmission to the drone for movement control. Further improvement can be made by incorporating an adaptive control algorithm to the drone’s control and reduction of window size to stabilize the trajectory followed by the drone.

## Discussion

In this study, we decoded eight active brain commands using hybrid EEG–fNIRS for BCI. The generated commands were tested using a quadcopter. To the best of the authors’ knowledge, there are only two previous studies that have tested their BCI schemes for control of a quadcopter in 3D space (LaFleur et al., 2013; Kim et al., 2014). Our work has an advantage over both studies, as we were able to control the quadcopter with a greater number of active commands. LaFleur et al. (2013) controlled the height and rotation of a quadcopter using motor imageries for the left, right, and both hands. However, in this case, the quadcopter was given a fixed forward speed. Therefore, this work did not give full quadcopter control to the user for navigation. Also, as it is impossible for some subjects to perform motor imagery (Vidaurre and Blankertz, 2010), their scheme is suited only to a specific set of users who can in fact perform it. Kim et al. (2014) integrated EEG with an eye tracker for generation of eight commands. Although their eye tracking was effective, an LED-based flash light was used to monitor the eye movement. As such, with the illuminating LED enhancing the contrast between the pupil and iris, it would be difficult to maintain quadcopter-controlling concentration for any significant span of time. In our case, there are no such drawbacks, as the necessary mental and eye movement commands are easy to generate. Also, our scheme yields more freedom to the user for drone control. It also includes a measure—specifically EEG/fNIRS integration—for avoidance of any miss-directional movement. A given EEG command has been matched with an opposing fNIRS command (see Figure 7). Thus, in the event that an EEG command is incorrectly classified and the quadcopter follows a wrong direction, fNIRS signals can be used to counteract that command.

Our proposed scheme for fNIRS classification incorporates features for both hemodynamics and initial dips. To the best of our knowledge, this is the first work to generate commands in a 0–2 s window for BCI. Whereas previously, different windows have been reported for fNIRS-based classification using fNIRS, in the current work, the smallest window size for classification was used. Although the reported optimal window size, which is to say, the size allowing for the highest classification accuracy, is 2–7 s (Naseer and Hong, 2013), the 0–2 s window size, albeit causing a decrease in accuracy, is still usable for BCI. Also, we used the mean, min, and peak values of ΔHbO for classification. Further investigation of feature selection and the use of adaptive algorithms will improve the results in both window size and classification.

Another advantage of the proposed scheme is its decoding of all four fNIRS activities from the prefrontal cortex. In previous research (Naseer and Hong, 2015a), four choices have been decoded using fNIRS in a 2–7 s window. However, in this case, only two classes were decoded from the prefrontal cortex, the other two commands having been generated using data from the motor cortex. Our proposed work is more advantageous for more users, as those suffering from locked-in syndrome cannot properly perform motor tasks. Another advantage of our work is the reduced command-generation time using fNIRS. This enables patients who are partially locked-in (with minor eye movements) to use eight commands in controlling a robot in online/real-time scenarios.

A previous fNIRS study (Hong and Santosa, 2016) proposed channel selection based on a *t-*value criterion. It classified four sound categories from the auditory cortex using the channels’ highest *t*-values. In our approach, we used the baseline as a reference for selection of channels. For classification, we selected, for four prefrontal activities, the common channels yielding a positive value after taking the difference between the peak value and the baseline. The drawback here is that only a limited number of channels can be selected. The algorithm can be further improved by adding the “difference of mean” for channel selection. In the comparison of our approach with *t-*value-based channel selection (see Table 4), most of the selected channels were common. It can be seen that the *t*-value-based scheme can identify more active channels. However, channel detection time also is an important factor for real-time applications, and our proposed scheme can identify the activated channels much quicker than the previous schemes. Thus, our method allows much room for further development in terms of command generation and real-time control.

**Table 4:** **Comparison of selected channels and time between the proposed method and the *t*-value-based method**.

Subjects	Selected channels			
	The proposed scheme	Selection time (s)	*t*-value-based scheme	Selection time (s)
1	4, 10	0.0005	4, 8, 10	0.172
2	4, 9, 10	0.0005	2, 3, 4, 9, 10, 11	0.185
3	3, 5, 7,11, 12, 15	0.0005	2, 3, 5, 7, 11, 15	0.203
4	2, 3, 5, 6, 11, 14	0.0005	2, 3, 5, 6, 9, 11, 14, 15	0.192
5	6, 7, 14, 15	0.0005	5, 6, 7, 14, 15, 16	0.195
6	6, 8–16	0.0005	1,5–16	0.198
7	1, 4, 5, 8, 15	0.0005	1, 2, 3, 4, 5, 8, 10	0.191
8	1, 2, 3, 4, 5, 8, 11, 14	0.0005	1, 2, 3, 4, 5, 8, 11–15	0.198
9	1, 2, 3, 4, 5, 6,10	0.0005	1, 2, 3, 4, 5, 8, 9, 10, 16	0.196
10	1, 2, 6, 7, 14	0.0005	1, 5, 6, 7, 9, 13, 14	0.172

A limitation of this method is the acquisition of activities using eye movement tasks. Although the use of eye movement for robot control has already been demonstrated to be effective (Ma et al., 2015), eye movements are related to motor activity, and so, it is difficult for motor-disabled patients to generate four EEG-based commands. The selection of different active tasks for EEG can improve the results. Another, fNIRS-related limitation of the proposed method is the variation in hemodynamic responses in subjects due to trial-to-trial variability (Hu et al., 2013). Granted, the proposed features (peak, mean, and min ΔHbO) might not yield the best performance for each subject in the 0–2 s window. However, this problem is not insoluble, and certainly, it will be addressed in further investigations into feature selection. Also, the use of adaptive algorithms promises improvement in fNIRS command generation time.

## Conclusion

In this study, we investigated the possibility of decoding eight commands from the frontal and prefrontal cortices by combining electroencephalography and functional near-infrared spectroscopy (fNIRS) for a BCI. Four EEG commands were generated by eye movements (two and three blinks as well as left/right and up/down movements), using the number of peaks and the mean value as features. In the case of fNIRS, we chose mental counting, mental arithmetic, mental rotation, and word formation tasks for the purpose of activity decoding. We selected a 0–2 s window to generate the commands using fNIRS signals. The signal mean, peak, and minimum values were used as features for incorporation of hemodynamic signals and initial dip features in the classifier. The obtained 76.5% accuracy indicates the possibility of classifying the activities in reduced windows. We tested the generated commands in a real-time scenario using a quadcopter. The movement coordinates of the quadcopter were updated using the hybrid EEG–fNIRS-based commands. The performed experiments served to demonstrate the BCI feasibility and potential applications of the proposed eight-command decoding scheme. Further research on better feature selection and minimization of time window for command generation can improve the controllability of the quadcopter. Moreover, the incorporation of adaptive algorithms for flight control along with brain signal decoding for stable flight can further strengthen the results.

## Author Contributions

MJK has conducted all the experiments, carried out the data processing, and made initial manuscript. K-SH has suggested the theoretical aspects of the current study and supervised all the process from the beginning. All the authors have approved the final manuscript.

## Conflict of Interest Statement

The authors declare that the research was conducted in the absence of any commercial or financial relationships that could be construed as a potential conflict of interest.
